# Comprehensive imaging evaluation of the aortic valve and root before aortic root surgery: a study comparing MDCT and TEE

**DOI:** 10.1186/s12872-024-04031-6

**Published:** 2024-07-16

**Authors:** Yuan Li, Shuai Zhang, Hongxia Qi, Xiaoyan Ma, Xiangyang Qian, Jing Sun

**Affiliations:** 1 Department of Cardiovascular Surgery, Fuwai Hospital, Chinese Academy of Medical Sciences & Peking Union Medical College/National Center for Cardiovascular Diseases, No. 167 North Lishi Road, Xicheng District, Beijing, 100037 China; 2 Department of Ultrasound, Fuwai Hospital, Chinese Academy of Medical Sciences & Peking Union Medical College/National Center for Cardiovascular Disease, Beijing, China; 3 Department of Radiology, Fuwai Hospittal, Chinese Academy of Medical Sciences & Peking Union Medical College/National Center for Cardiovascular Diseases, Beijing, China

**Keywords:** Aortic root surgery, Multidetector coomputed tomography, Transeophageal echocardiography

## Abstract

**Objective:**

To investigate the accuracy and consistency of MDCT and TEE in the preoperative assessment of aortic root surgery.

**Methods:**

From January 2021 to September 2022, 118 patients who underwent aortic root surgery were included in this study. All patients underwent high-quality preoperative MDCT and TEE examinations, and the examination results were independently measured and assessed by two senior radiologists or ultrasound specialists. Bland–Altman analysis and Pearson correlation testing were employed to assess the correlation and consistency between MDCT and TEE. These analyses were then compared with actual intraoperative measurement data.

**Results:**

Among all the patients, 73 (61.86%) had tricuspid aortic valve (TAV), and 45 (38.14%) had bicuspid aortic valve (BAV). A comparison between the TEE and MDCT measurements showed that for the annulus diameter, the area-derived diameter had the best correlation and agreement. For the sinus of Valsalva diameter, the circumference-derived diameter was optimal. However, for the STJ diameter, the minimum cross-sectional diameter showed the best agreement with TEE. In contrast, measurements of geometric height showed a weaker correlation and agreement.

**Conclusion:**

Contrast-enhanced MDCT can be a valuable tool for perioperative evaluation in aortic root surgery, with good correlation, consistency, and feasibility when compared to TEE. The choice of MDCT measurement methodology, specifically area-derived and circumference-derived diameter, proved to be more accurate than other methods. Further research is required to enhance the understanding of aortic valve repair and associated imaging techniques.

**Supplementary Information:**

The online version contains supplementary material available at 10.1186/s12872-024-04031-6.

## Introduction

Aortic valve repair has become a common surgical approach for treating aortic valve or root disease in selected patients, with the advantages of preserving the native valve and no postoperative anticoagulation requirement compared to aortic valve replacement. Two basic surgical approaches were proposed in the 1990s: remodeling and reimplantation, along with numerous other modifications [1–6]. After years of clinical practice, aortic valve repair has achieved excellent clinical outcomes, with low reoperation rates and high survival rates for two decades [7]. In addition, aortic valve repair is associated with a significant reduction in valve-related death and major bleeding events [8].

The modern approach to aortic valve repair focuses on the functional anatomy of the aortic root, encompassing both root dimensions and aortic cusp geometry. By utilizing a functional classification of aortic root pathology, this approach facilitates the rational application of valve-sparing surgical procedures, achieving excellent outcomes. [9, 10]. The ratio of the cusp geometry correlation is relatively stable in the aortic root, which serves in valve-sparing surgery [11]. Thus, accurate preoperative assessments of the aortic valve and root are vital.

Contrast-enhanced multidetector computed tomography (MDCT) and transesophageal echocardiography (TEE) are commonly used to evaluate the aortic valve and root. Echocardiography is one of the most reliable methods for evaluating the aortic root, is convenient, allows real-time observation of valve pathophysiology and is dynamic. TEE offers a distinct advantage in preoperative aortic valve surgery by providing detailed identification of the aortic regurgitation (AR) jet location. TEE can predict complications such as fenestration rupture or perforation of the aortic valve cusp before surgery. These insights are crucial for planning interventions like cusp repair, including reinforcement of the free margin or pericardial patch repair. However, in the measurement of aortic root geometry, MDCT MDCT complements TEE by accurately measuring various parameters of aortic root geometry. This information is essential for predicting the size of the prosthetic graft or valve required during surgery. On the other hand, because of the valve movement, in the assessment of leaflets, TEE is free from leaflet movement. Together, TEE and MDCT offer a complementary approach to preoperative evaluation in aortic root surgery, enhancing the precision and success of surgical planning. However, Studies have shown that TEE tends to underestimate aortic annulus measurements when compared to MDCT [12]. Regarding the methodology of measurement, clinical data publications consider the leading edge-to-leading edge (L-L) as the most standard convention [13]. On the other hand, the American College of Cardiology and American Heart Association (AHA) guidelines suggest the use of inner-edge to inner-edge (I-I) CT or MR when performing CT or MR in patients with known or suspected aortic disease and the use of outer-edge to outer-edge (O-O) CT or MR in patients with aortic wall abnormalities [14]. However, the American Society of Echocardiography considers leading edge-to-leading edge methods to be conventional methods [15]. Thus, several controversies remain between the consistency and measurement methodology used in MDCT and TEE.

Therefore, this observational, prospective study aimed to compare the agreement and feasibility of the aortic valve geometry parameters measured by MDCT and TEE and compare their accuracy with intraoperative data. In addition, we aimed to determine the best measurement methodology for aortic root imaging.

## Materials and methods

### Study population

This study was conducted from January 2021 to September 2022. A total of 146 patients with aortic valve or root disease who required MDCT and TEE before aortic root surgery (including aortic valve repair, valve-sparing aortic root surgery and the Bentall procedure) were prospectively recruited at a single tertiary center. The exclusion criteria included (a) known contrast allergies (*n* = 2), (b) severe renal dysfunction who could not undergo contrast-CT (*n* = 5), (c) poor ultrasound or CT imaging (*n* = 9), and (d) inability to hold the breath for 20 s or a heart rate greater than 70 bpm after the administration of a beta-adrenergic blocking agent. (*n* = 5), (e) atrial fibrillation during examination (*n* = 3), and (f) refusal to provide informed consent (*n* = 4). Thus, a total of 118 patients who underwent MDCT and TEE were recruited. This study was approved by the Ethics Committee of Fuwai Hospital (Approval No. 2020 − 1282). All patients signed informed consent forms.

### Study design

**Fig. 1:**
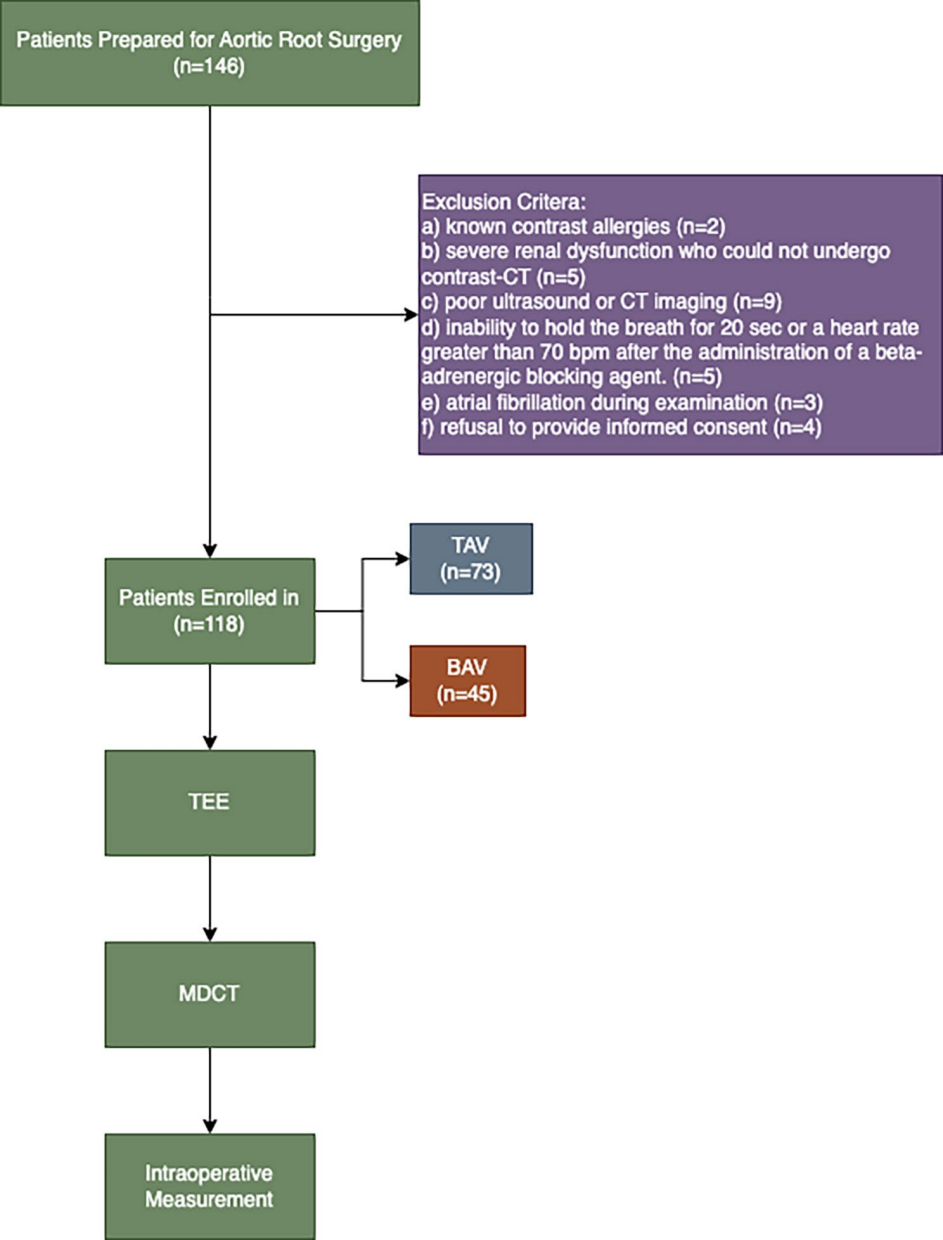
Research flowchart

All patients underwent 2 TEE examinations (1 preoperative to assess the aortic root and 1 postoperative to assess valve function) and 1 ECG-gated MDCT examination preoperatively. After initial evaluation by preoperative TTE, patients who were scheduled for aortic root surgery were referred to the Department of Radiology to complete the ECG-gated MDCT examination and the Department of Ultrasound to complete the TEE examination for further evaluation. The interval between the TEE and MDCT examinations was < 1 week. All patients underwent immediate TEE following the completion of surgery. The flowchart of this study is shown in Fig. 1.

### TEE

**Fig. 2:**
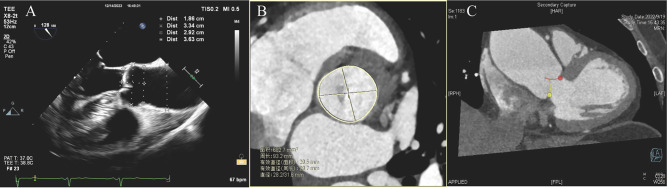
Images of the aortic root and geometric parameters on MDCT and TEE.

All patients underwent TEE preoperatively using a 5.0-mHZ, 128-element, multiplane ultrasound unit (Phillips, Bothell, WA, USA) by two experienced echocardiographers, and intra- and interobserver agreement were evaluated. The aortic root parameters were measured at different levels in the following order: aortic annulus, sinuses of Valsalva (SVA), Sino tubular junction (STJ), geometry height and ascending aorta, as showed in Fig. 2A. All parameters were measured by two individual observers.

### MDCT

Contrast-enhanced 64-slice spiral multidetector computed tomography (Lightspeed Volume CT, GE healthcare, Little Chalfont, UK) was performed in all patients with a collimation of 64 × 0.625 mm and a tube voltage of 100 to 120 kV. Contrast media (50–60 ml) was administered via an arm vein at a speed of 5 ml/s, followed by an injection of saline (40 ml, 5 ml/s). During the examination, all patients’ heart rates were under 70 bpm. Those whose heart rate exceeded 70 bpm were administered a beta-adrenergic blocking agent. All parameters (including I-I, L-L and O-O) were measured at the 75% phase of the R-R interval. Figure 2B and C shows the diameters of the aortic root, including the circumference-derived diameter (D-circ), area-derived diameter (D-area), maximum diameter (D-max) and minimum diameter (D-min), were calculated via four different methods.

### Intraoperative measurement

All patients underwent aortic root surgery via a standard, median sternotomy approach. After the aortic valve was exposed, the size of the aortic annulus was assessed. The sizing obturator was applied to the annulus to detect the annulus diameter when it fit the annulus completely.A sterile surgical compass was utilized to measure the diameter of STJ and aortic root. A caliper designed by Schäfers was used to measure the aortic cusp geometry intraoperatively; the longer edge was placed at the lowest point of the central point, and the shorter edge was pushed to the free margin while the curve accommodates the free margin [9]. By straightening the aortic valve leaflets, the geometric height is directly measured using a sterile ruler.

### Statistical analysis

All the data were tested for normality by Shapiro–Wilk’s test. Continuous variables are expressed as the means and standard deviations. Differences between groups were tested by Student’s t test for normally distributed variables and the Mann‒Whitney U test for nonnormally distributed variables. Categorical variables are expressed as percentages, and differences between groups were tested by the chi-square test or Fisher’s exact test. Bland–Altman analysis and correlation analyses were performed to analyze the consistency between MDCT and TEE. Statistical significance was defined as *p* < 0.05 (two-tailed). IBM SPSS statistics 26.0 software was used for the data analysis.

## Results

**Table 1:** Patient demographic information and clinical characteristics

Parameters	All (*n* = 118)	TAV (*n* = 73)	BAV (*n* = 45)	*p* value
Age, years(+/-SD)	39.29, 13.78	35.45,12.05	41.66,14.31	0.0167
Male, (n%)	107(90.68)	63(86.3)	44(97.77)	0.079
Height, m	177.0,10.34	175.6,8.747	177.8,11.19	0.27
Weight, kg	76.44,16.21	76.89,18.04	75.71,12.87	0.7029
BMI	24.33,4.427	24.17,4.695	24.58,3.993	0.6245
Body Surface Area(m^2^)^*^	1.905,0.2449	1.916,0.247	1.887,0.1882	0.5436
Hypertension, n (%)	41(34.74)	28(38.35)	13(28.89)	0.3953
Hypercholesterolemia (%)	15(12.71)	8(10.96)	7(15.56)	0.466
Diabetes (%)	20(16.94)	11(15.07)	9(20)	0.488
NYHA class, n (%)				
I	35(29.66)	22(30.13)	13(28.89)	0.8854
II	66(55.93)	41(51.16)	25(55.56)	0.9484
III	16(13.55)	9(12.33)	7(15.56)	0.6190
IV	1(0.85)	1(1.37)	0	> 0.99
LVEF, %	58.14, 7.32	56.89, 6.773	58.90, 7.581	0.1471
Aortic Valve Regurgitation, n (%)				
-None - Mild	28(23.73)	20(27.39)	8(17.78)	0.2712
- Mild –Moderate	14(11.86)	9(12.33)	5(11.11)	0.5716
- Moderate - Severe	76(64.40)	44(60.27)	32(71.11)	0.2324
Marfan, n(%)	35(29.66)	30(41.09)	5(11.11)	0.0005
Congenital Heart Disease, n(%)	4(3.39)	3(4.11)	1(22.22)	0.5716
Type A Dissection, n(%)	5(4.24)	5(6.85)	0	0.1550
Surgery				
David I	71	54	17	< 0.0001
David II	5	2	3	0.3681
AVP	21	3	18	< 0.0001
Bentall	21	14	7	0.6173

*Body surface area was calculated = (height in cm)^(0.725) * (weight in kg)^(0.425) * 0.007184

AVP: Aortic valve plasty

**Table 2:** MDCT and TEE measurements of aortic root geometry

		MDCT (I-I)	TEE(I-I)	*r*	*p*	bias	LOA
Annulus Diameter	D-circ	29.40,3.703	29.01,3.031	0.8502	< 0.001	0.4672	7.661
	D-area	28.64,3.549	29.01,3.031	0.8664	< 0.001	-0.2778	6.951
	D-max	32.12,4.170	29.01,3.031	0.8178	< 0.001	3.119	9.523
	D-min	25.87,3.603	29.01,3.031	0.8203	< 0.001	-3.131	8.083
Sinuses of Valsava Diameter	D-circ	49.73,7.893	49.26,8.376	0.8932	< 0.001	0.4678	14.851
	D-area	48.00,7.498	49.26,8.376	0.8810	< 0.001	-1.262	15.542
	D-max	50.72,7.749	49.26,8.376	0.8808	< 0.001	1.454	15.612
	D-min	44.37,7.384	49.26,8.376	0.7929	< 0.001	-4.892	20.216
STJ Diameter	D-circ	46.51,10.76	43.67,9.832	0.8868	< 0.001	2.847	19.522
	D-area	45.96,10.51	43.67,9.832	0.8918	< 0.001	2.295	18.727
	D-max	47.87,10.83	43.67,9.832	0.8807	< 0.001	4.207	20.131
	D-min	44.24,10.17	43.67,9.832	0.8857	< 0.001	0.5678	4.792
Geometry height							
TAV							
-RCC		18.20,2.672	19.20,2.259	0.5901	< 0.001	-0.7941	8.54
-LCC		19.40,2.133	19.83,2.585	0.5352	< 0.001	0.6338	8.727
-NCC		21.56,2.773	20.07,2.381	0.5890	< 0.001	1.645	8.966
BAV							
-Fusion cusp		17.00,3.498	17.15,2.008	0.3895	0.0142	-0.4256	12.395
-Nonfusion cusp		21.50,2.873	21.98,2.180	0.6675	< 0.001	-0.5357	8.62

**Fig. 3:**
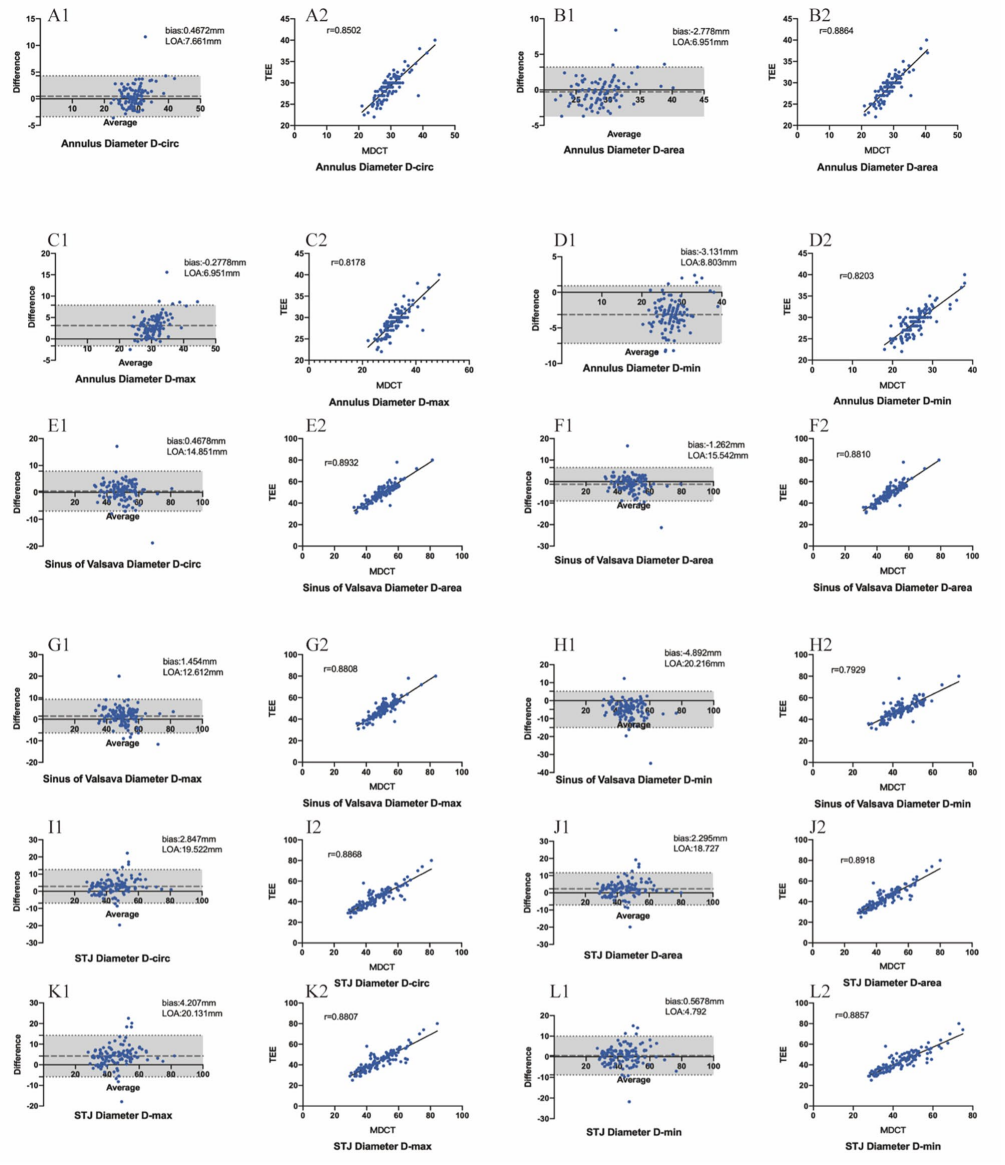
Scatter plot of Bland‒Altman plots and correlation between MDCT and TEE measurements. **A**) Comparison between the MDCT D-circ method and TEE for measuring the annulus diameter, **B**) comparison between the MDCT D-area method and TEE for measuring the annulus diameter, **C**) comparison between the MDCT D-max method and TEE for measuring the annulus diameter, **D**) comparison between the MDCT D-min method and TEE for measuring the annulus diameter, **E**) comparison between the MDCT D-circ method and TEE for measuring the sinus of Valsalva diameter, **F**) comparison between the MDCT D-area method and TEE for measuring the sinus of Valsalva diameter, **G**) comparison between the MDCT D-max method and TEE for measuring the sinus of Valsalva diameter, **H**) comparison between the MDCT D-min method and TEE for measuring the sinus of Valsalva diameter, **H**) comparison between the MDCT D-circ method and TEE for measuring the STJ diameter, **I**) comparison between the MDCT D-area method and TEE for measuring the STJ diameter, **J**) comparison between the MDCT D-max method and TEE for measuring the STJ diameter, **K**) comparison between the MDCT D-min method and TEE for measuring the STJ diameter

**Fig. 4:**
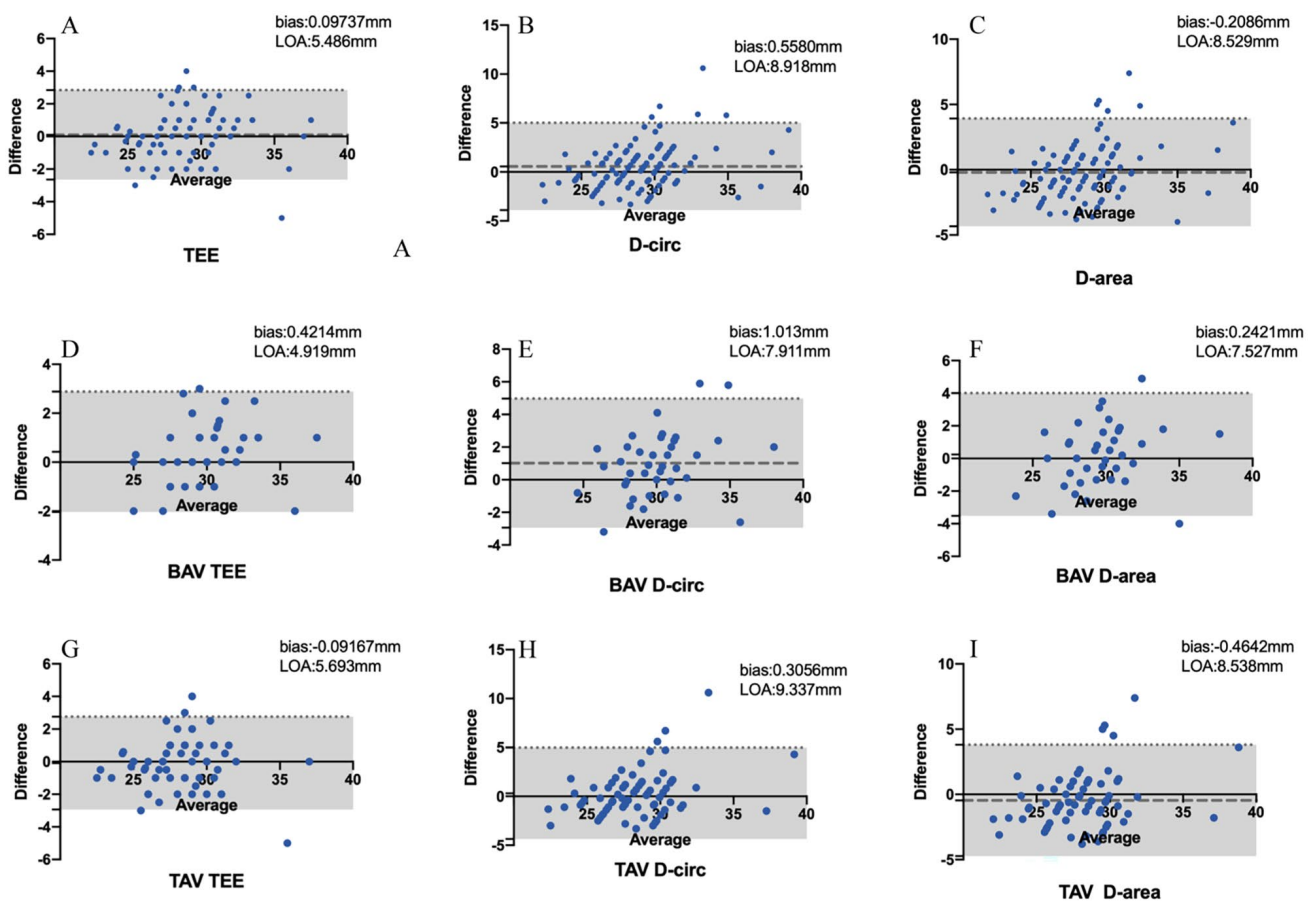
Comparison between MDCT and TEE measurements of the annulus diameter and intraoperative measurement data. **A**) Comparison of TEE measurements with intraoperative data, **B**) comparison of MDCT D-circ method data with intraoperative data, **C**) comparison of MDCT D-area method data with intraoperative data. **D**), **E**), and **F**) Comparison of measurement results in BAV patients using TEE, MDCT D-circ, and MDCT D-area methods. **G**), **H**), and **I**) Comparison of measurement results in TAV patients using TEE, MDCT D-circ, and MDCT D-area methods

### Demographic data

The demographic data and clinical characteristics of the patients are shown in Table 1. A total of 118 patients were included in the study. Regarding the morphology of the aortic valve, 73 (61.86%) patients had tricuspid aortic valve (TAV) morphology, and 45 (38.14%) had bicuspid aortic valve (BAV) morphology. Thirty-five patients (29.66%) had Marfan syndrome, 4 (3.39%) had congenital heart disease, and 5 (4.24%) had type A aortic dissection. Among all the patients, 28 (23.73%) had no regurgitation or mild regurgitation, 35 (29.66%) had mild to moderate regurgitation, and 76 (64.40%) had moderate to severe regurgitation as assessed by TEE.

### Measurement of aortic root geometry via different imaging techniques

Table 2 reports the TEE and MDCT results. According to the definition, the TEE measurements of the annulus diameter, SVA diameter and STJ diameter (I-I) were 29.01 ± 3.031 mm, 49.26 ± 49.26 mm and 43.67 ± 9.832 mm, respectively. Considering the different measurement approaches used for MDCT, D-circ and D-area were closer to TEE than were D-max and D-min: annulus diameter (D-circ: 29.40 ± 3.703 mm vs. D-area: 28.64 ± 3.549 mm vs. D-max: 32.12 ± 4.170 mm vs. D-min: 25.87 ± 3.603 mm); SVA diameter (D-circ: 49.73 ± 7.893 mm vs. D-area: 48.00 ± 7.498 mm vs. D-max: 50.72 ± 7.749 mm vs. D-min: 44.37 ± 7.384 mm); and STJ diameter (D-circ: 46.54 ± 10.76 mm vs. D-area: 45.96 ± 10.51 mm vs. D-max: 47.87 ± 10.83 mm vs. D-min: 44.24 ± 10.27 mm). Geometry height measured by TEE and MDCT revealed slight differences in all cusps for both the TAV and BAV: RCC (TEE: 19.20 ± 2.259 mm vs. MDCT: 18.20 ± 2.672 mm); LCC (TEE: 19.83, 2.585 mm vs. MDCT: 19.40 ± 2.133 mm); NCC (TEE: 20.07 ± 2.381 mm vs. MDCT: 21.56 ± 2.773 mm); fusion cusp (TEE: 17.15 ± 2.008 vs. MDCT: 17.00 ± 3.498); and nonfusion cusp (TEE: 21.98 ± 2.180 mm vs. MDCT: 21.50 ± 2.873).

### Differences and consistency between MDCT and TEE

Figure 3 shows the scatter plot of the correlation and Bland‒Altman plots comparing measurements obtained by the TEE and MDCT techniques (I‒I). For the annulus diameter, the best correlation and agreement were acquired by the area-derived diameter (D-area) using the cross-sectional area of the aortic annulus (*r* = 0.8664, LOA = 6.951 mm, bias=-0.2778 mm). For the sinus of Valsalva diameter, the best performance method was acquired by the circumference-derived diameter (D-circ) using the cross-sectional area of the aortic annulus (*r* = 0.8932, LOA = 14.851 mm, bias = 0.4678 mm). However, for the STJ diameter, the minimum cross-sectional diameter showed the best agreement compared to that of TEE (*r* = 0.8857, LOA = 4.792 mm, bias = 0.5678 mm), while the D-area showed the best correlation (*r* = 0.8918, LOA = 18.727, bias = 2.295). For the geometric height, the correlation and agreement were weaker than those for root diameter (TAV: RCC vs. LCC vs. NCC: *r* = 0.5901, t = 0.5352, *r* = 0.5890; BAV: fusion cusp vs. nonfusion cusp: *r* = 0.3895, *r* = 0.6675).

### Annulus diameter accuracy compared to the intraoperative data

The Bland‒Altman plots comparing the TEE and MDCT results with the intraoperative data are shown in Fig. 4. For all patients, TEE (bias: 0.09737 mm, LOA: 5.486 mm) was more accurate than MDCT. However, in BAV patients, the D-area MDCT method showed better accuracy and agreement than did TEE (MDCT: bias: 0.2427 mm, LOA: 7.527 mm vs. TEE: bias: 0.4214 mm, LOA: 4.919 mm).

## Discussion

The primary findings of this study are as follows: (1) In the preoperative assessment of the aortic root, MDCT demonstrates superior correlation and consistency compared to TEE, particularly because of the greater accuracy of the D-area and D-circ methods; (2) For irregular annulus, such as in BAV patients, MDCT exhibits significant advantages in measurement; (3) High-quality MDCT images are valuable for preoperative geometric height measurements.

Previous studies have commonly employed echocardiography to evaluate the aortic valve and aortic root [16]. As the most commonly utilized diagnostic modality for preoperative assessment of the aortic root, echocardiography provides comprehensive anatomical information on the root and assesses aortic valve function. It enables real-time visualization of the physiological status of the aortic valve and aortic root for surgeons, facilitating the evaluation of aortic valve regurgitation mechanisms, the assessability of aortic valve repair, and the formulation of corresponding surgical strategies [14]. Our study employed Bland‒Altman analysis and correlation analysis to compare the relationships between MDCT and TEE measurements of the aortic annulus, sinus of Valsalva, and STJ. The findings revealed a high level of consistency between MDCT and TEE. Consequently, MDCT has emerged as a viable method for the preoperative assessment of the aortic root.

MDCT has significant advantages in measuring the diameter of the aortic root. Although there is controversy in guideline recommendations, there is a consensus within the industry on the methodology for measurements. The I-I method is most commonly employed in MDCT measurements when contrast is administered, as the wall itself is scarcely visible. Conversely, the O-O method is utilized for noncontrast-enhanced scans [17].

Echocardiography is also commonly employed for the measurement of effective height and geometric height. However, in comparison to echocardiography, MDCT offers superior spatial resolution, providing a significant advantage in measuring root dimensions such as geometric height and effective height. As a result, there is a growing body of research utilizing MDCT for the assessment of aortic root dimensions [18]. Our study revealed a high degree of similarity between MDCT and TEE results in assessing geometric height, demonstrating a high level of reliability. However, in measuring effective height and free margin length, both MDCT and TEE have certain limitations, and the majority of studies still rely on intraoperative measurements.

In comparison to TAV patients, BAV patients often exhibit an elliptical or irregular valve annulus. In a previous study, the BAV annulus was measured separately at the level of the sinus of the valsava because it was not possible to measure three cusp-to-commissure distances in the BAV [17]. Our study revealed that MDCT has a distinct advantage for annulus diameter measurements in BAV patients. Furthermore, our findings indicate a greater correlation between MDCT and TEE in BAV patients. Compared to intraoperative measurements, MDCT exhibited greater accuracy, with the highest precision observed in D-area measurements, followed by D-circ measurements.

The main limitations of this study are as follows: (1) our study used a 64-slice spiral multidetector scanner, as opposed to a 128-slice scanner, which entails much more strict control of heart rate and a high dose of radiation; (2) in our study, all MDCT measurements were taken at 75% of the cardiac cycle, which is consistent with previous and current guidelines that favor the use of measurements in the systolic phase; though, there is no evidence supporting better consistency between diastolic measurements and intraoperative findings; and (3) compared to previous series from Western or other Asian countries, our cohorts are very young and cannot be applied to other scales. (4) This study was unable to assess the status of the aortic root in patients with impaired renal function who could not undergo MDCT and those with suboptimal image quality due to elevated heart rate during examination or inability to breath-hold. (5) The exclusion of a healthy control group in this study diminishes the generalizability of the research findings.

## Conclusion

Compared with TEE, contrast-enhanced MDCT imaging of the aortic root dimensions can be performed for the perioperative evaluation of aortic root surgery, with good correlation, consistency and feasibility, especially in patients with irregular annuli, such as the BAV. MDCT has greater advantages. In MDCT measurements, the area-derived diameter and circumference-derived diameter are more accurate than the minimum and maximum cross-sectional diameters.

### Electronic supplementary material

Below is the link to the electronic supplementary material.

Supplementary Material 1

## Data Availability

Data is provided within the manuscript or supplementary information files.
